# Safety, tolerability and pharmacokinetics of the fibroblast growth factor receptor inhibitor AZD4547 in Japanese patients with advanced solid tumours: a Phase I study

**DOI:** 10.1007/s10637-016-0416-x

**Published:** 2017-01-10

**Authors:** Hideo Saka, Chiyoe Kitagawa, Yoshihito Kogure, Yasuo Takahashi, Koshi Fujikawa, Tamotsu Sagawa, Satoru Iwasa, Naoki Takahashi, Taro Fukao, Catherine Tchinou, Dónal Landers, Yasuhide Yamada

**Affiliations:** 1 Department of Medical Oncology, Nagoya Medical Center, 4-1-1 Sannomaru, Naka-ku, Nagoya, Aichi 460-0001 Japan; 2 Department of Gastroenterological Medicine, Hokkaido Cancer Center, Hokkaido, Japan; 3 Department of Medical Oncology, Gastrointestinal Medical Oncology Division, National Cancer Center Hospital, Tokyo, Japan; 4 R&D, AstraZeneca KK, Osaka, Japan; 5 AstraZeneca, Macclesfield, UK

**Keywords:** AZD4547, FGFR, Japanese, Phase I, Safety, Pharmacokinetics

## Abstract

*Background* AZD4547 is a potent, oral, highly selective fibroblast growth factor receptor (FGFR) inhibitor in clinical development for treating tumours with a range of FGFR aberrations, including FGFR mutations, amplifications and fusions. *Methods* This open-label, Phase I, multicentre study (NCT01213160) evaluated the safety, pharmacokinetics, and preliminary antitumour efficacy (RECIST v1.1) of AZD4547 monotherapy in Japanese patients with advanced solid tumours. Part A was a dose-escalation part; Part B was a dose-expansion part in patients with FGFR-amplified tumours, confirmed by fluorescence in situ hybridization. *Results* Thirty patients enrolled in Part A (dose range: 40 mg twice daily [bid] to 120 mg bid; 160 mg once daily [qd]), four in Part B (80 mg bid). No dose-limiting toxicities were observed and maximum tolerated dose was not determined. Most common adverse events (AEs; any grade) were: dysgeusia (50% of patients); stomatitis (41%); diarrhoea (38%); hyperphosphataemia (38%); dry mouth (35%). Common grade ≥3 AEs were nausea (12% of patients) and neutropenia (9%). No complete or partial responses were observed: 21/30 patients had stable disease ≥4 weeks in Part A, and 1/4 patients had stable disease ≥10 weeks in Part B. Following single and multiple dosing, absorption rate appeared moderate; peak plasma concentrations generally occurred 3–4 h post-dose, then declined biphasically with terminal half-life ~30 h. Steady state was reached by day 8. Compared with single dosing, plasma concentrations were, on average, 2.4- and 3.3- to 5.4-fold higher after qd and bid dosing, respectively. *Conclusions* AZD4547 was well tolerated in Japanese patients, with best response of stable disease ≥4 weeks.

## Introduction

Fibroblast growth factor receptors (FGFRs) are transmembrane receptor tyrosine kinases with varied biological roles in regulating angiogenesis, cell proliferation, differentiation, migration and survival. Altered FGFR signalling has the potential to drive mitogenic, invasive, anti-apoptotic and pro-angiogenic cells and has been increasingly implicated in a range of solid tumour types, including breast cancer (BC), high-grade bladder cancer, non-small-cell lung cancer (NSCLC) and gastric cancer (GC), as well as haematological malignancies. Of the five known FGFRs found in humans, FGFR1–4 are characterized by extracellular immunoglobulin-like and intracellular tyrosine kinase domains, whereas the atypical FGFR5 (also known as fibroblast growth factor receptor-like 1) lacks the cytoplasmic tyrosine kinase domain; consequently, its role is less understood. There are several mechanisms underlying the misregulation of FGFRs in neoplastic disease, including activating mutations in FGFRs [1, 2], FGFR gene amplification [2–6], FGFR chromosomal translocations [7–9], alternative splicing of FGFRs [10], and altered autocrine and paracrine signalling at FGFRs via FGF [2].

AZD4547 is a potent, oral, highly selective inhibitor of FGFR1–3 with proven antitumour properties from preclinical studies [11–15], including work in FGFR2-amplified GC xenografts that demonstrated complete and prolonged tumour regression in several AZD4547-treated animals [12]. An initial Phase I study in a Western population indicated that AZD4547 monotherapy has an acceptable safety profile in patients with several tumour types (NCT00979134). During this study, a partial response (PR) was observed following AZD4547 treatment in a patient with FGFR1-amplified squamous NSCLC. Stable disease was experienced by 4/21 additional patients (19.0%), three of whom had confirmed FGFR amplification status (squamous NSCLC, *n* = 1; bladder cancer, *n* = 1; BC, *n* = 1) [16]. These data suggest a potential association between FGFR amplification status and clinical benefit with AZD4547 therapy.

It is thought that FGFRs mediate angiogenesis through their synergistic role with vascular endothelial growth factor receptors (VEGFRs). The success of bevacizumab, a monoclonal antibody that became the first approved anti-VEGF therapy, has given rise to several anti-angiogenic therapies, most notably, a group of oral tyrosine kinase inhibitors (TKIs) targeting VEGFR. Although these drugs, which include sunitinib [17, 18], sorafenib [19–22], pazopanib [23, 24] and cediranib [25–28], have demonstrated promising results in patients with advanced cancer, resistance generally develops following an initial clinical response, and patients experience relapse. Preclinical data have demonstrated that tumours with resistance to anti-VEGF therapies can over-express FGFs, and there is clinical evidence indicating that disease progression following bevacizumab treatment is preceded by an increase in levels of basic FGF (bFGF) [29, 30]. Elevated bFGF levels were also significantly associated with shorter overall survival in cediranib-treated patients [30, 31]. These data suggest that inhibition of FGFRs, together with direct antitumour activity, may play a role in preventing resistance to anti-angiogenic drugs [29].

This Phase I study (NCT01213160) was designed to evaluate the safety and tolerability, appropriate dosing, pharmacokinetic (PK) profile, and preliminary antitumour effects of AZD4547 when administered in Japanese patients with advanced solid malignancies.

## Methods

### Patients

Eligible patients had confirmed solid malignancies for which standard therapies did not exist or were no longer effective, a World Health Organization (WHO) performance status of 0–1, and a life expectancy of at least 12 weeks. Previous preclinical data have revealed pharmacodynamic effects on cartilage and growing bones following treatment with another FGFR inhibitor [32]. In order to ensure that maturation of the skeleton is complete upon entry into this study, eligible patients must be aged ≥25 years. Exclusion criteria included: any chemotherapy, immunotherapy, or anticancer agents ≤3 weeks prior to study entry; major surgery or radiotherapy ≤4 weeks prior to study entry; nitrosourea or mitomycin C ≤6 weeks prior to study entry; any unresolved toxicities from previous treatments exceeding Common Terminology Criteria for Adverse Events (CTCAE) grade 1 (excluding alopecia). Specific cardiac- and ophthalmologically related exclusion criteria included: clinically important electrocardiogram (ECG) abnormalities; QT interval ≥470 ms; history or evidence of retinal pigmented epithelial detachment; history or evidence of age-related macular degeneration. Other exclusion criteria included: spinal cord compression; brain metastases; severe or uncontrolled systemic disease; inadequate bone marrow reserves or organ function. The study was approved by the independent ethics committee, research ethics board or institutional review board at each centre and complied with the International Conference on Harmonisation’s Harmonised Tripartite Guidelines for Good Clinical Practice, the Declaration of Helsinki and local laws. All patients provided written informed consent.

### Study design

**Fig. 1:**
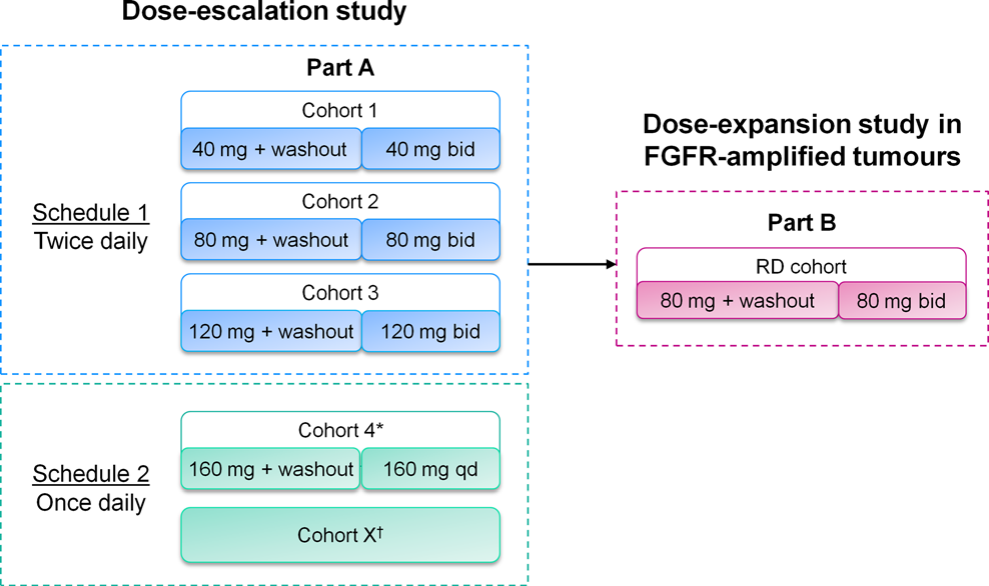
AZD4547 Japanese Phase I study design. Part A was a dose-escalation study with a 5- to 10-day washout period followed by bid dosing. Part B was a dose-escalation study in patients with FGFR-amplified tumours with an RP2D of 80 mg bid. *Cohort 4 dose was based on PK modelling data and was consistent with the latest tolerated exposures from AZD4547 bid dosing in Western patients [16], as well as emerging safety data from Japanese patients (this study); ^†^In schedule 2, it was planned that dose assessment could extend over multiple cohorts; however, no cohorts exceeded the 160 mg qd dosing level due to emerging data from the study in Western patients and a decision from the clinical project team. RP2D, recommended Phase II dose

This Phase I, open-label, Japanese, multicentre study was conducted in two parts (Fig. 1). Part A was a dose-escalation phase. Single oral dosing (40 mg; 80 mg; 120 mg) was followed by a 1-week washout period. Multiple oral dosing was delivered in 21-day cycles according to two treatment schedules: schedule one (40 mg twice daily [bid]; 80 mg bid; 120 mg bid) and schedule two (160 mg once daily [qd]). Part B was an expansion phase that evaluated a recommended dose (RD) of 80 mg bid in FGFR-amplified tumours. FGFR amplification was determined by central fluorescence in situ hybridization (FISH) testing of archival tumour samples. This RD was determined using both emerging data from Part A and existing data from the study in Western patients (NCT00979134) [16].

A ‘rolling six’ design was used, with a minimum of three evaluable patients per cohort [33]. If one patient experienced a dose-limiting toxicity (DLT), additional patients were enrolled up to a maximum of six evaluable patients. DLTs were evaluated during the washout period and the first 21-day treatment schedule. These were defined as any toxicity not attributable to the disease under investigation, including haematological toxicity of CTCAE grade ≥4, non-haematological toxicity of CTCAE grade ≥3, and any other toxicity that was clinically significant, did not respond to supportive care and resulted in discontinuation of dosing. If two or more evaluable patients experienced a DLT, this dosing level was considered as non-tolerable. It was planned that the maximum tolerated dose (MTD) would be defined either as the dosing level below the non-tolerated dose or such that a dose between the non-tolerated dose and the last tolerated dose may be investigated. Patients who tolerated AZD4547 treatment and received clinical benefit were permitted to continue treatment until they experienced progressive disease or withdrew consent.

### Study objectives

The primary objective of this study was to evaluate the safety and tolerability of oral AZD4547 in Japanese patients with advanced solid malignancies. Secondary objectives included defining the MTD and/or a tolerable RD, characterizing the PK properties following both single and multiple dosing of AZD4547, and exploring the preliminary antitumour activity of AZD4547.

### Assessments

#### Safety and tolerability

Safety and tolerability were assessed during study treatment and until 28 days after the final dose. Adverse events (AEs) were evaluated according to CTCAE (version 4.0), and dose interruptions and reductions were recorded. Laboratory findings and vital signs were analysed. Cardiac monitoring (echocardiogram [ECHO] and ECG) and ophthalmic assessments were also conducted.

#### Pharmacokinetic assessments

Blood samples for PK analysis were collected pre-dose and at defined intervals up to 96 h following single dosing, and up to 24 h following multiple dosing. Urine samples were also collected during the 24 h after multiple dosing in order to perform urinary PK assessments. For multiple dosing, the 80 mg bid dosing level was evaluated using combined data from patients in both Part A and Part B, as FGFR amplification status was unlikely to have a significant impact on PK. Concentrations of AZD4547 in human plasma and urine were determined using a validated high-performance liquid chromatography–tandem mass spectrometry method at PRA International (Assen, The Netherlands). PK parameters were analysed by standard non-compartmental methods using WinNonlin software (Pharsight Corporation, Mountain View, CA, USA).

#### Efficacy

Tumour assessments were performed according to Response Evaluation Criteria in Solid Tumors version 1.1 (RECIST v1.1) at baseline, on day 21 of the first treatment cycle, then every 6 weeks after the start of treatment for 12 weeks, and thereafter every 12 weeks (±1 week) until discontinuation of study treatment or withdrawal of consent.

#### Statistics

No formal hypothesis-led statistical analysis was performed. Safety, tolerability, PK data and efficacy were summarized using descriptive statistics. Analysis sets for safety and efficacy contained all patients who received ≥1 dose of AZD4547. PK analysis included all patients who provided blood samples.

## Results

### Patient characteristics

Between 5 November 2010 and 22 November 2012, 30 Japanese patients were enrolled in Part A of this study (male, *n* = 16; female, *n* = 14) and four in Part B (male, *n* = 3; female, *n* = 1). All patients received at least one dose of AZD4547 and were evaluable for safety, PK, and efficacy analyses. A summary of patient characteristics is given in Table 1. The mean age of patients was 62.3 years (range 30–78 years) in Part A and 70.8 years (range 64–76 years) in Part B. The major primary tumour locations were lung (33.3% in Part A; 25.0% in Part B), breast (16.7%; 25.0%), and stomach (13.3%; 50.0%). The majority of patients (93.3%) in Part A and all patients in Part B had metastatic disease, with the respiratory system and lymph nodes as the most commonly reported disease sites. At entry into this study, most patients had received prior radiotherapy (96.7% in Part A; 100% in Part B) and all had received previous chemotherapy, with 26.7% and 25.0% of patients having received ≥3 lines of previous chemotherapy in Parts A and B, respectively.

**Table 1:** Patient demographics and baseline characteristics

	AZD4547 dose					
	Part A					Part B
	40 mg bid	80 mg bid	120 mg bid	160 mg qd	Total Part A	80 mg bid
	(*N* = 3)	(*N* = 6)	(*N* = 6)	(*N* = 15)	(*N* = 30)	(*N* = 4)
Sex, n (%)						
Female	1 (33.3)	4 (66.7)	2 (66.7)	7 (46.7)	14 (46.7)	1 (25.0)
Male	2 (66.7)	2 (33.3)	4 (33.3)	8 (53.3)	16 (53.5)	3 (75.0)
Median age, years	49	63.5	61.5	66.0	63.5	71.5
(range)	(41–73)	(30–76)	(47–76)	(51–78)	(30–78)	(64–76)
WHO performance status, n (%)						
0	3 (100)	5 (83.3)	3 (50.0)	7 (46.7)	18 (60.0)	2 (50.0)
1	0 (0)	1 (16.7)	3 (50.0)	8 (53.3)	12 (40.0)	2 (50.0)
Local/metastatic sites, n (%)						
Local only	0 (0)	0 (0)	1 (16.7)	1 (6.7)	2 (6.7)	0
Local/metastatic	3 (100)	6 (100)	5 (83.3)	14 (93.3)	28 (93.3)	4 (100)
Common primary tumour types, n (%)						
Bile duct	0 (0)	0 (0)	0 (0)	1 (6.7)	1 (3.3)	0 (0)
Bladder	0 (0)	0 (0)	0 (0)	1 (6.7)	1 (3.3)	0 (0)
Breast	0 (0)	4 (66.7)	0 (0)	1 (6.7)	5 (16.7)	1 (25.0)
Caecum	1 (33.3)	0 (0)	0 (0)	0 (0)	1 (3.3)	0 (0)
Colon	1 (33.3)	1 (16.7)	0 (0)	0 (0)	2 (6.7)	0 (0)
Colorectal	0 (0)	0 (0)	0 (0)	1 (6.7)	1 (3.3)	0 (0)
Lung	0 (0)	1 (16.7)	3 (50.0)	6 (40.0)	10 (33.3)	1 (25.0)
Oesophagus	0 (0)	0 (0)	0 (0)	1 (6.7)	1 (3.3)	0 (0)
Pancreas	0 (0)	0 (0)	1 (16.7)	0 (0)	1 (3.3)	0 (0)
Rectal	0 (0)	0 (0)	0 (0)	1 (6.7)	1 (3.3)	0 (0)
Stomach	0 (0)	0 (0)	1 (16.7)	3 (20.0)	4 (13.3)	2 (50.0)
Thymus	1 (33.3)	0 (0)	0 (0)	0 (0)	1 (3.3)	0 (0)
Urachus	0 (0)	0 (0)	1 (16.7)	0 (0)	1 (3.3)	0 (0)
Prior therapy, n (%)						
Chemotherapy	2 (66.7)	6 (100)	6 (100)	15 (100)	29 (96.7)	4 (100)
Other systemic anticancer therapy	2 (66.7)	2 (33.3)	1 (16.7)	6 (40.0)	11 (36.7)	1 (25.0)
Radiotherapy	3 (100)	6 (100)	6 (100)	15 (100)	30 (100)	4 (100)
Hormonal/immunotherapy	0 (0)	1 (16.7)	0 (0)	2 (13.3)	3 (33.3)	0 (0)

### Safety and tolerability

#### Dose escalations

During the dose-escalation phase in Part A, AZD4547 dosing was escalated in three cohorts in schedule one (40 mg bid; 60 mg bid; 120 mg bid). Based on the emerging safety profile, the safety review committee authorized the initiation of schedule two, a once-daily dose, in a fourth cohort (160 mg qd). However, based on emerging data from the study in Western patients [16] and a decision from the clinical project team, the qd dose regimen was not escalated to 240 mg.

No DLTs were observed across any of the four cohorts examined and given the decision not to titrate beyond the once-daily schedule of 160 mg qd, the MTD was not determined for Japanese patients in this study. Instead, the recommended dose of 80 mg bid for assessment in Part B was determined based on safety data from Part A of this study alongside the data in Western patients [16]. No DLTs were observed with the RD of 80 mg bid in Part B.

All patients had discontinued the study by the data cut-off date (16 August 2013). The most common reasons for discontinuation in Part A were disease progression in 14/30 patients (46.7%), AEs in 9/30 patients (30.0%; 80 mg bid, *n* = 3; 160 mg qd, *n* = 6), and death in 1/4 patients (25.0%; 160 mg qd). All patients (80 mg bid, *n* = 4) in Part B discontinued AZD4547 following disease progression.

#### Summary of AEs

Overall, 32/34 patients (94.1%) experienced at least one AE following AZD4547 treatment (Table 2); the AEs of 30/34 patients (88.2%) were considered by the investigator to be causally related to AZD4547. The most frequently reported AEs (≥20%) in Part A were dysgeusia in 14 patients (46.7%), diarrhoea in 12 (40.0%), stomatitis in 12 (40.0%), hyperphosphataemia in 11 (36.7%), dry mouth in 10 (33.3%), dry skin in nine (30.0%), nausea in eight (26.7%), detachment of retinal pigment epithelium in seven (23.3%), vomiting in six (20.0%), malaise in six (20.0%), nail discolouration in six (20.0%), and pruritus in six (20.0%). The most common AEs (≥50%) in Part B were dysgeusia in three patients (75.0%) and stomatitis, hyperphosphataemia, dry mouth, nausea, and decreased appetite, which were all present in two patients each (50.0%). Three patients (10.0%) experienced an AE of CTCAE grade ≥3 in Part A (80 mg bid); these were judged to be causally related to the study treatment in one patient (3.3%). Three patients (75.0%) experienced an AE of CTCAE grade ≥3 in Part B; however, these AEs were not deemed to be treatment related. Overall (*N* = 34), the most common CTCAE grade ≥3 AEs were neutropenia in three patients (8.8%), nausea in two (5.8%), and decreased appetite in three (8.8%). Three SAEs were experienced by two patients (6.7%) in Part A and one patient (25.0%) in Part B. All patients with SAEs required hospitalization. Only one SAE, decreased appetite and nausea, was deemed to be causally related to AZD4547 treatment (Part A; 80 mg bid). Two further SAEs, stomatitis (Part A; 80 mg bid) and decreased appetite (Part B; 80 mg bid), were not considered to be causally related to treatment with AZD4547. One death occurred during the study period (160 mg qd) following disease progression; the study investigators concluded that this death was not causally related to AZD4547 treatment.

**Table 2:** Summary of AEs occurring in ≥20% of all patients, AEs of grade ≥3 occurring in ≥5% of all patients, and SAEs for each cohort

	AZD4547 dose					
	Part A					Part B
	40 mg bid (*N* = 3)	80 mg bid (*N* = 6)	120 mg bid (*N* = 6)	160 mg qd (*N* = 15)	Total Part A (*N* = 30)	80 mg bid (*N* = 4)
Patients with AE of any grade, n (%)	3 (100)	6 (100)	6 (100)	14 (93.3)	29 (97.6)	4 (100)
Dysgeusia	0 (0)	2 (33.3)	5 (83.3)	7 (50.0)	14 (46.7)	3 (75.0)
Diarrhoea	0 (0)	2 (33.3)	5 (83.3)	5 (33.3)	12 (40.0)	1 (25.0)
Stomatitis	1 (33.3)	4 (66.7)	4 (66.7)	3 (20.0)	12 (40.0)	2 (50.0)
Hyperphosphataemia	0 (0)	1 (16.7)	3 (50.0)	7 (50.0)	11 (36.7)	2 (50.0)
Dry mouth	0 (0)	3 (50.0)	2 (33.3)	5 (33.3)	10 (33.3)	2 (50.0)
Dry skin	1 (33.3)	2 (33.3)	2 (33.3)	4 (26.7)	9 (30.0)	0 (0)
Nausea	1 (33.3)	2 (33.3)	1 (16.7)	4 (26.7)	8 (26.7)	2 (50.0)
Detachment of retinal pigment epithelium	0 (0)	0 (0)	0 (0)	7 (50.0)	7 (23.3)	1 (25.0)
Vomiting	0 (0)	2 (33.3)	2 (33.3)	2 (13.3)	6 (20.0)	1 (25.0)
Malaise	0 (0)	2 (33.3)	2 (33.3)	2 (13.3)	6 (20.0)	1 (25.0)
Decreased appetite	1 (33.3)	1 (16.7)	1 (16.7)	2 (13.3)	6 (20.0)	2 (50.0)
Patients with CTCAE grade ≥3 event, n (%)	0 (0)	3 (50.0)	0 (0)	0 (0)	3 (10.0)	3 (75.0)
Neutropenia	0 (0)	2 (33.3)	0 (0)	0 (0)	2 (6.7)	1 (25.0)
Nausea	0 (0)	2 (33.3)	0 (0)	0 (0)	2 (6.7)	0 (0)
Decreased appetite	0 (0)	1 (16.7)	0 (0)	0 (0)	1 (3.3)	2 (50.0)
Stomatitis	0 (0)	1 (16.7)	0 (0)	0 (0)	1 (3.3)	0 (0)
Pneumonia	0 (0)	0 (0)	0 (0)	0 (0)	1 (3.3)	1 (25.0)
Increased alanine aminotransferase	0 (0)	1 (16.7)	0 (0)	0 (0)	1 (3.3)	0 (0)
Decreased appetite	0 (0)	1 (16.7)	0 (0)	0 (0)	1 (3.3)	0 (0)
Hypoglycaemia	0 (0)	0 (0)	0 (0)	0 (0)	1 (3.3)	1 (25.0)
Patients with SAE grade ≥3 event, n (%)	0 (0)	0 (0)	2 (33.3)	0 (0)	2 (6.7)	1 (25.0)
Nausea	0 (0)	0 (0)	1 (16.7)	0 (0)	1 (3.3)	0 (0)
Stomatitis	0 (0)	0 (0)	1 (16.7)	0 (0)	1 (3.3)	0 (0)
Decreased appetite	0 (0)	0 (0)	1 (16.7)	0 (0)	1 (3.3)	1 (25.0)

*SAE* serious adverse event

#### Dose interruptions and reductions

Nine patients (30.0%) in Part A reported dose interruptions following AEs, and 13/30 patients (43.3%) experienced dose reductions, of whom 12 (40.0%) had reductions following AEs, most commonly detachment of retinal pigment epithelium or other retinal disorders, as well as hyperphosphataemia and dizziness. One patient (25.0%) in Part B had a dose interruption as a result of an AE of decreased appetite, and two patients (50.0%) had dose reductions after reporting AEs of retinal detachment, nausea, and hypoglycaemia. The mean actual treatment duration was 80.1 days in Part A and 36.0 days in Part B. The mean relative dose intensity was 87% in Part A and 83.5% in Part B.

#### Dose discontinuation

In Part A, 9/30 patients (30.0%) had an AE leading to discontinuation of the study drug, and these AEs were considered causally related to the study drug by the investigator. None of the patients in Part B had AEs leading to discontinuation. Retinal events led to study-drug discontinuation in 7/34 patients (20.5%) and all patients recovered.

#### Other safety observations

Treatment-related increases in blood phosphate levels were observed in 11 patients (36.7%) in Part A and two patients (50.0%) in Part B, with median change in phosphate levels from baseline ranging from –0.16 to 0.79 mmol/L in the 80 mg bid cohort (combined from Parts A and B) to –0.29 to 0.72 mmol/L in the 160 mg qd cohort. Time to onset ranged from 9 to 24 days. All except one patient received treatment with fosrenol in accordance with the management guidelines for hyperphosphataemia and recovered. No clinically relevant changes in vital or physical signs were observed. One patient (120 mg bid) with a normal ECG at baseline experienced an abnormal ECG with AZD4547 treatment; however, this was not considered to be clinically relevant. Three patients experienced a decrease in left ventricular ejection fraction (LVEF) of ≥10 percentage points and three patients experienced an absolute LVEF value of <55%; however, no patients were reported to have fulfilled both criteria simultaneously and, consequently, these changes were not considered to be clinically relevant. Grade 1 and 2 decreases in platelet counts were observed in 13/30 patients (43.3%), and only in the 160 mg qd cohort. All other clinical laboratory observations were comparable between dosing levels. A trend in mean-value increase for transaminases and blood creatinine was observed, which consisted mainly of a one-grade shift.

### Pharmacokinetics

**Fig. 2:**
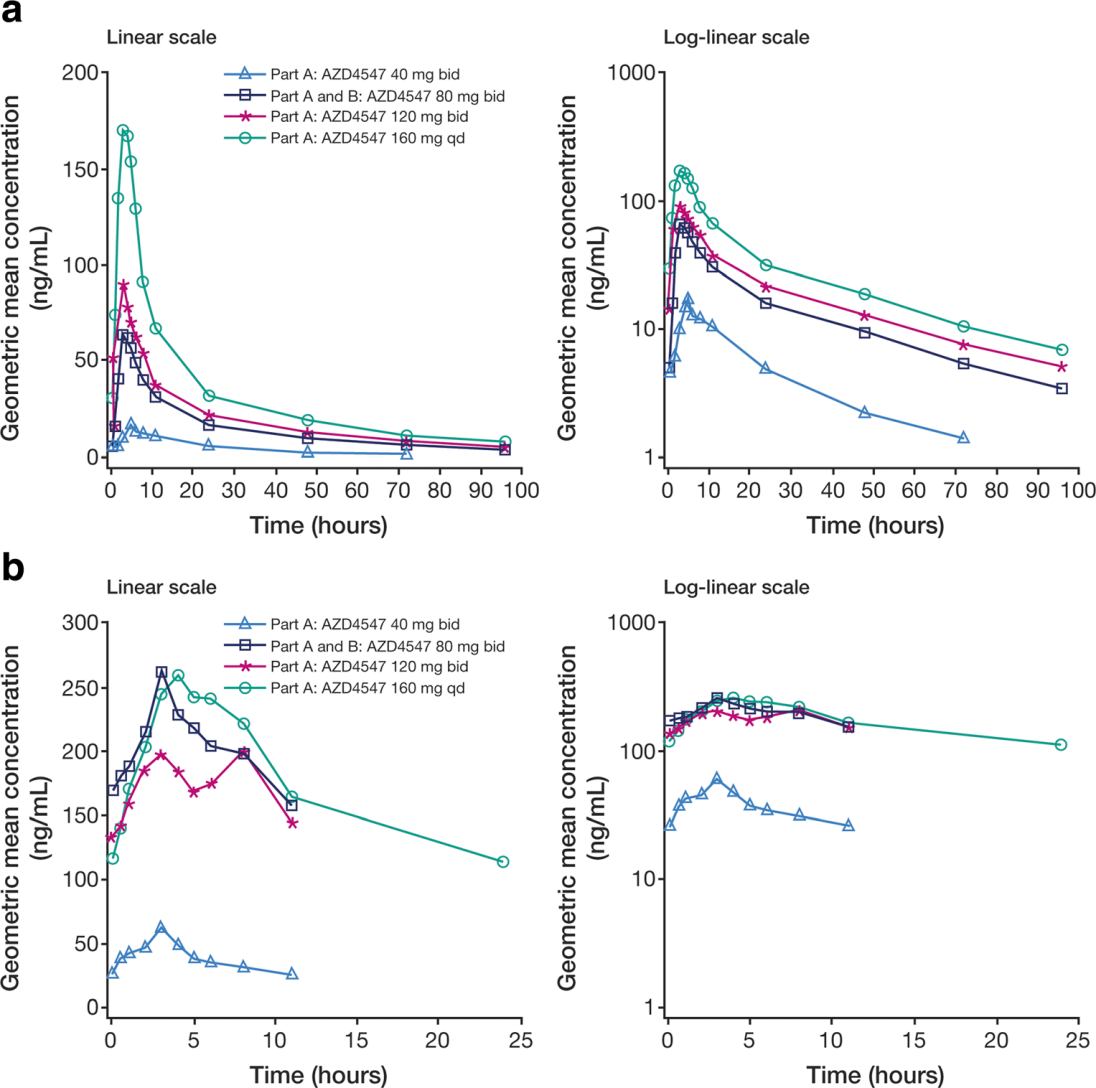
Plasma concentration–time profiles of AZD4547 after **a** single dosing and **b** multiple dosing. Geometric mean plasma concentrations are shown against time for the dosing levels 40 mg bid, 80 mg bid (combined from cohorts dosed at the 80 mg bid level across both Parts A and B), 120 mg bid, and 160 mg qd

Following single dosing, AZD4547 plasma levels were quantifiable across all investigated dosing levels. The mean plasma concentration–time profiles for single and multiple dosing are shown in Fig. 2. A summary of PK parameters is given in Table 3. Median time to maximum plasma concentration (t_max_) ranged from 2.9 to 4.0 h across the dose levels of 40–160 mg. After reaching maximum plasma concentration (C_max_), AZD4547 concentrations declined biphasically, with a mean terminal half-life (t_1/2λz_; ± standard deviation [SD]) ranging from 22.4 (±7.21) to 33.5 (±7.49) h. The ratio of the area under the plasma concentration–time curve from time 0 to infinity (AUC) to that from time 0 to time of last measurable concentration (AUC_0–t_) was >0.87, indicating that the sampling scheme used had reliably captured the plasma concentration–time profiles. The percentage coefficient of variation (CV%) values for C_max_ and AUC were 54.0–142% and 53.3–117%, respectively, across the dosing levels. Dose-normalized C_max_ and AUC values were 9.58–21.4 ng/mL and 0.61–1.35 h · ng/mL, respectively, for the dosing levels tested. Mean (±SD) oral clearance (CL/F) ranged from 57.8 (±27.3) to 116 (±77.0) L/h and was independent of dose across the dosing range of 80–160 mg.

**Table 3:** Plasma and urinary PK parameters of AZD4547 (PK analysis set)

Parameter	Summary statistic	AZD4547 single dosing				AZD4547 multiple dosing			
		Part A	Part A and Part B	Part A	Part A	Part A	Part A and Part B	Part A	Part A
		40 mg bid (*N* = 3)	80 mg bid (*N* = 10)	120 mg bid (*N* = 6)	160 mg qd (*N* = 15)	40 mg bid (*N* = 3)	80 mg bid (*N* = 10)	120 mg bid (*N* = 6)	160 mg qd (*N* = 15)
AUC, h · ng/mL^a^	n	3	9	6	14	3	8	5	12
	Mean^b^ (CV%)	394.1 (67.17)	1539 (53.28)	2058 (116.6)	3417 (66.96)	456.6 (10.46)	2379 (56.09)	2072 (42.08)	4134 (61.04)
Dose-normalized AUC, h · ng/mL^a^	n	3	9	6	14	3	8	5	12
	Mean^b^ (CV%)	9.584 (67.17)	19.24 (53.28)	17.15 (116.6)	21.36 (66.96)	11.41 (10.46)	29.74 (56.09)	17.27 (42.08)	25.84 (61.04)
AUC_0–t_, h · ng/mL^a^	n	3	10	6	15	3	8	5	12
	Mean^b^ (CV%)	353.9 (71.52)	1452 (50.71)	1794 (124.1)	3055 (63.53)	408.3 (11.06)	2099 (56.97)	1808 (40.80)	4134 (60.87)
C_max_, ng/mL^a^	n	3	10	6	15	3	8	5	12
	Mean^b^ (CV%)	24.30 (54.07)	88.41 (81.41)	99.78 (141.5)	216.2 (68.30)	67.81 (23.00)	272.8 (60.13)	215.8 (36.43)	302.9 (65.89)
Dose-normalized C_max_, ng/mL^a^	n	3	10	6	15	3	8	5	12
	Mean^b^ (CV%)	0.6075 (54.07)	1.105 (81.41)	0.8315 (141.5)	1.351 (68.30)	1.695 (23.00)	3.410 (60.13)	1.799 (36.43)	1.893 (65.89)
CL/F, ng/mL^a^	n	3	9	6	14	3	8	5	12
	Mean^b^ (SD)	116.0 (77.04)	82.13 (71.61)	57.94 (50.75)	57.94 (27.34)	87.93 (9.373)	37.90 (19.85)	61.66 (23.69)	44.55 (24.51)
t_1/2λz_, h	n	3	9	6	14	–	–	–	–
	Mean^d^ (SD)	22.42 (7.215)	28.82 (5.198)	33.46 (7.492)	27.96 (4.214)	–	–	–	–
t_max_, h^a^	n	3	10	6	15	3	8	5	12
	Median	4.000	3.000	2.990	2.930	2.920	2.950	3.930	3.960
R_AC_^c^	n	–	–	–	–	3	8	5	12
	Mean^d^ (SD)	–	–	–	–	3.336 (1.984)	4.870 (2.421)	5.378 (5.432)	2.423 (1.555)
Tc	n	–	–	–	–	3	8	5	11
	Mean^d^ (SD)	–	–	–	–	1.372 (1.014)	1.914 (0.8593)	1.610 (1.409)	1.372 (0.8796)
fe, %	n	–	–	–	–	2	8	5	12
	Mean^d^ (SD)	–	–	–	–	3.921 (0.8283)	3.662 (1.248)	4.133 (1.676)	3.779 (2.477)
CL_R_, L/h	n	–	–	–	–	2	8	5	12
	Mean^d^ (SD)	–	–	–	–	3.229 (0.5796)	1.248 (0.4322)	2.319 (0.6347)	1.383 (0.5867)

^a^Parameters at steady state for multiple dosing; ^b^Mean geometric mean; ^c^Ratio of multiple-dose AUC_ss_ to AUC_0–12h_ [bid dosing] or AUC_0–24h_ [qd dosing]); ^d^Mean arithmetic mean

AZD4547 plasma levels were quantifiable across all levels and time points for multiple dosing, and steady state (ss) was reached by day 8. The CV% range for C_ss,max_ was 23.0–65.9%, and the CV% range for AUC_ss_ was 10.5–61.0%. Dose-normalized values for C_ss,max_ and AUC_ss_ were 1.60–3.41 ng/mL and 11.4–29.7 h · ng/mL across the different multiple dosing levels. Median t_max_ ranged from 2.9 to 4.0 h post-dose, in line with the data from single dosing. Mean (±SD) CL_ss_/F ranged from 37.9 (±19.9) to 87.9 (±9.37) L/h, which were lower than the CL/F values for single doses. The mean (±SD) accumulation ratio (R_AC_; ratio of multiple-dose AUC_ss_ to single-dose AUC_0–12h_) was 3.34 (±1.98), 4.87 (±2.42), and 5.34 (±5.43), respectively, for 40 mg, 80 mg, and 120 mg bid dosing. The mean (±SD) value of R_AC_ (ratio of multiple-dose AUC_ss_ to single-dose AUC_0–24h_) for the 160 mg qd cohort was 2.42 (±1.56). The mean (±SD) values of temporal change (Tc; ratio of multiple-dose AUC_ss_ to single-dose AUC) were 1.37 (±1.01), 1.75 (±0.86), 1.61 (±1.41) and 1.37 (±0.88), respectively, for 40 mg, 80 mg, 120 mg bid and 160 mg qd dosing.

Urinary PK data were available for 27 patients (25/30 patients in Part A; 2/4 patients in Part B). The mean (±SD) steady-state fraction of the AZD4547 dose excreted in urine (fe) was 3.92% (±0.828%), 3.66% (±1.25%), 4.13% (±1.68%) and 3.78% (±2.48%) for 40 mg, 80 mg, 120 mg bid and 160 mg qd dosing, respectively, suggesting that urinary excretion of AZD4547 is dose independent. Mean (±SD) steady-state renal clearance (CL_R_) values were variable at 3.23 (±0.58), 1.25 (±0.43), 2.32 (±0.63) and 1.38 (±0.59) L/h, respectively, for 40 mg, 80 mg, 120 mg bid and 160 mg qd dosing, but showed no dose-dependency trend.

### Preliminary efficacy

Complete responses and partial responses, according to RECIST v1.1, were not observed; however, stable disease (≥4 weeks’ duration) was observed in 21 patients (70.0%; 40 mg bid, *n* = 3; 80 mg bid, *n* = 3; 120 mg bid, *n* = 5; 160 mg qd, *n* = 10) in Part A, with one patient (25.0%; 80 mg bid) continuing to experience stable disease at 10 weeks in Part B. Except for one patient in Part A with a non-evaluable response, all remaining patients showed disease progression. Post-baseline target lesion measurements were available in 25/30 patients in Part A and all patients in Part B; the median percentage change in the sum of the diameters was 5.9% (range –6.8% to 48.9%) in Part A and 3.0% (range –16.3% to 23.4%) in Part B.

## Discussion

The FGFR pathway is involved in key cellular processes necessary for survival and differentiation. Accordingly, aberrant FGFR signalling has significant oncogenic potential. This Phase I study is the first to evaluate the safety and tolerability of AZD4547 in a population of Japanese patients with advanced solid malignancies for which no standard or effective treatment exists. The characteristics of this study population were comparable to those of the intended target population for AZD4547.

### Safety profile

Overall, AEs during AZD4547 treatment were generally mild to moderate and reversible upon withdrawal of treatment, as has been observed previously [16]. No DLTs were observed in our study and the drug was not titrated to an MTD as a result of emerging safety data from the Phase I study conducted in Western patients [16]. In the study in Western patients, AZD4547-treatment-related DLTs of renal failure, elevated liver enzymes, hyperphosphataemia, and mucositis were observed at the dose range 20–200 mg bid [16]. The absence of causally related DLTs in our Japanese population may, therefore, be explained by the lower dosing levels, despite body mass in the Japanese patients being smaller than in the Western patients. An RD of 80 mg bid was determined based on the combined safety data from our study and from the study in Western patients [16]; this RD was evaluated during the expansion phase.

Results of the expansion phase showed that the RD of 80 mg bid was well tolerated in Japanese patients with FGFR-amplified tumours (as determined by FISH). Consistent with data from Western patients, the most common AEs in our study were gastrointestinal disorders, dryness, hyperphosphataemia, and eye disorders and included diarrhoea, stomatitis, dry mouth and skin, nausea, dysgeusia, and detachment of retinal pigment epithelium [16, 34, 35]. Similar safety findings have been reported for other selective FGFR receptors [36–38]. One of the safety concerns for selective FGFR inhibitors from preclinical toxicity studies has been hyperphosphataemia, caused by loss of FGF23 signalling, resulting in calcification of tissues [2, 32]. All phosphate-related events in our study were of CTCAE grade ≤3 and were controllable with therapeutic interventions. Taken together with available clinical data from AZD4547 and other FGFR inhibitors, this indicates that hyperphosphataemia with FGFR inhibitors is generally manageable in humans [16, 37].

### Pharmacokinetics

The PK findings were generally consistent between AZD4547 single and multiple dosing. Following bid dosing in three cohorts (40 mg; 80 mg; 120 mg) and qd dosing in one cohort (160 mg), steady state was achieved by day 8, and the accumulation ratio was consistent with the prediction from single-dose t_1/2λz_. Dose-normalized PK parameters for single and multiple doses were similar across all dosing levels; however, the small number of patients in each cohort and the variability between plasma concentration–time plots make dose proportionality difficult to establish. Tc tended to be close to or slightly higher than unity, suggesting that there were no notable time-dependent changes in PK upon multiple dosing. A relatively small proportion of AZD4547 was excreted in the urine unchanged (3.8–4.1% of the dose), suggesting that urinary excretion may be a minor route of AZD4547 elimination if AZD4547 absorption is good in humans. The results reported here are the first published PK data for AZD4547 in any patient population, and it would therefore be interesting to compare our PK findings with subsequent PK data that may emerge from the ongoing clinical development of AZD4547.

### Efficacy and comparisons with other FGFR inhibitors

The best response following AZD4547 treatment in this study was stable disease for ≥4 weeks in 70% of patients, with one BC patient experiencing stable disease at 10 weeks. Previous efficacy data from the Western population showed a best response of PR (80 mg bid) for ≥12 weeks in one patient with FGFR1-amplified squamous NSCLC [16]; stable disease was also observed in 4/21 patients (19%), three of whom (75%) had confirmed FGFR amplification.

Efficacy data from clinical studies of other selective FGFR inhibitors have also been reported. In a Phase I study of BGJ398 (Novartis), PRs were observed in 2/17 evaluable patients with lung squamous cell carcinoma, with durations of 8 and 3 months [39]. It is important to note that this study population was selected on the basis of their FGFR amplification status. In an extended cohort of the same study, 8/25 patients with previously treated advanced/metastatic urothelial carcinoma (UC) and FGFR3 alterations had PRs, with one unconfirmed complete response [40]. Published findings from a Phase I trial of the selective FGFR inhibitor JNJ-42756493 (Johnson & Johnson) have shown PRs in 4/23 evaluable patients [36]. All patients demonstrating a PR had *FGFR2* or *FGFR3* translocations, and tumour types were reported as glioblastoma, UC, and endometrial cancer. Phase II studies of FGFR inhibitors are ongoing in different tumour types harbouring FGFR gene alterations. These include assessment of AZD4547 at the RD of 80 mg bid in FGFR2-amplified GC and FGFR1-amplified BC (NCT01457846; NCT01795768) [35, 41], BGJ398 in patients with advanced FGFR-altered colangiocarcinoma [38], and JNJ-42756493 in patients with metastatic or unresectable UC with FGFR gene alterations [42]. Selective FGFR inhibitors may also have potential in combination with other agents [43, 44].

Further investigation is required to establish the treatment settings in which this new class of drugs can provide the most meaningful clinical benefit. The initial expectation was that selection of patients by screening for FGFR amplification may identify responsive patients; however, patients with FGFR gene amplification have responded inconsistently to FGFR inhibitors [16, 45]. A key focus for development of selective FGFR inhibitors will therefore be to determine how aberrations in FGFR may be predictive of a response to treatment and incorporate appropriate predictive biomarkers into patient stratification. Taking this into consideration, the ongoing Phase Ib BISCAY biomarker-directed multidrug umbrella study (NCT02546661) will allocate patients to AZD4547 treatment based on FGFR3 mutations or FGFR1–3 fusions [46].

Several non-selective, multi-targeted TKIs have been licensed that can act as FGFR inhibitors, including pazopanib, lenvatinib, ponatinib, regorafenib, and nintedanib. In addition to FGFRs, these compounds have activity against a wide range of targets, including VEGFR1–3 and platelet-derived growth factor [47, 48], and have demonstrated clinical benefit for the treatment of several tumour types, such as renal cell carcinoma [23, 24], soft tissue sarcoma [49, 50], thyroid cancer [51], metastatic colorectal cancer [52], chronic myeloid leukaemia [53] and NSCLC [54, 55]. However, cardiac toxicity has been reported as a widespread AE with multi-targeted TKIs and is thought to be a dose-dependent, on-target effect related to the inhibition of VEGFR [56]. It is therefore useful to note that no significant cardiac toxicity was reported with AZD4547 treatment during this study, and cardiac toxicity has not been highlighted as a concern from previous studies with selective FGFR inhibitors [16, 35, 37, 39].

### Summary

AZD4547 was well tolerated in this Phase I study and no DLTs were reported. Based on safety data from Part A, and taking into account previous data in Western patients [16], the recommended dose was determined as 80 mg bid. Further investigation is required to establish the treatment settings in which this new class of drugs can provide the most meaningful clinical benefit.
